# Characterization and phylogenetic analysis of the complete mitochondrial genome of *Clavulina* sp. (Cantharellales: Clavulinaceae)

**DOI:** 10.1080/23802359.2020.1790327

**Published:** 2020-07-23

**Authors:** Maoling Tan, Gang Zhao

**Affiliations:** Key Laboratory of Coarse Cereal Processing, Ministry of Agriculture and Rural Affairs, College of Food and Biological Engineering, Chengdu University, Chengdu, PR China

**Keywords:** Ectomycorrhiza, mitochondrial genome, phylogenetic analysis, evolution, taxonomy

## Abstract

In this study, the complete mitochondrial genome of *Clavulina* sp. was sequenced and assembled. The complete mitochondrial genome of *Clavulina* sp. contains 20 protein-coding (PCG) genes, 2 ribosomal RNA (rRNA) genes, and 25 transfer RNA (tRNA) genes. The total size of the *Clavulina* sp. complete mitochondrial genome is 31,816 bp, with the GC content of 27.72%. Phylogenetic analysis indicated that the mitochondrial genome of *Clavulina* sp. exhibited a close relationship with that of the genus *Cantharellus*.

The genus *Clavulina* is an ectomycorrhizal fungal group distributed in many countries (Olariaga et al. 2009; Henkel et al. 2011). Most of species from the *Clavulina* genus are edible, and some species also show medicinal values (Khaund and Joshi 2014; Deo et al. 2019). Dozens of species have been described in genus *Clavulina* (Thacker and Henkel 2004; Uehling et al. 2012a, 2012b). Limited and varied morphological characteristics make it difficult to identify and classify *Clavulina* species accurately only by morphology (Thacker and Henkel 2004; Olariaga et al. 2009; Uehling et al. 2012a, 2012b). Mitochondrial genome is widely used to analyze the evolution and phylogeny of species (Li, He, et al. 2020; Wang et al. 2020; Li et al. 2020). However, up to now, no mitochondrial genome of *Clavulina* species has been published. The mitochondrial genome of *Clavulina* sp. will promote the understanding of the evolution, phylogeny, and taxonomy of this important ectomycorrhizal fungal group.

The specimen (*Clavulina* sp.) was collected from a mountain in Chuxiong, Yunnan, China (101.41 E; 25.12 N). The specimen was stored in Culture Collection Center of Chengdu University (No. Clasp08). The complete mitochondrial genome of *Clavulina* sp. was assembled according to previously described methods (Li, Xiang, et al. 2019; Wang et al. 2020). The total genomic DNA of *Clavulina* sp. was extracted using a Fungal DNA Kit D3390-00 (Omega Bio-Tek, Norcross, GA). A Gel Extraction Kit (Omega Bio-Tek, Norcross, GA) was used to purify the extracted total DNA. We stored the purified DNA in Chengdu University (No. DNA_ Clasp08). Sequencing libraries were constructed with the purified genomic DNA using a NEBNext^®^ Ultra™ II DNA Library Prep Kit (NEB, Beijing, China). We conducted whole genomic sequencing (WGS) of *Clavulina* sp. using the Illumina HiSeq 2500 Platform (Illumina, SanDiego, CA). The mitochondrial genome of *Clavulina* sp. was *de novo* assembled using SPAdes version 3.9.0 (Bankevich et al. 2012). We obtained the complete mitogenome of *Clavulina* sp., and then annotated it according to the methods described by Li, Chen, et al. (2018), Li, Ren, et al. (2019), and Li, Wang, et al. (2018).

The complete mitochondrial genome of *Clavulina* sp. is 31,816 bp in length, with the base composition as follows: A (34.94%), T (37.32%), G (14.38%), and C (13.35%). The complete mitochondrial genome of *Clavulina* sp. contains 20 protein-coding genes, 2 ribosomal RNA genes (*rns* and *rnl*), and 25 transfer RNA (tRNA) genes. To investigate the phylogenetic positions of *Clavulina* sp., we constructed a phylogenetic tree for 20 species. The phylogenetic tree was constructed using the Bayesian analysis (BI) method based on the combined 14 core protein-coding genes according to methods described by Li, Wang, Jin, Chen, Xiong, Li, Liu, et al. (2019), Li, Wang, Jin, Chen, Xiong, Li, Zhao, et al. (2019) and Li, Yang, et al. (2020). As shown in the phylogenetic tree (Figure 1), the mitochondrial genome of *Clavulina* sp. exhibited a close relationship with that of the genus *Cantharellus* (Li, Liao, et al. 2018).

**Figure 1.**
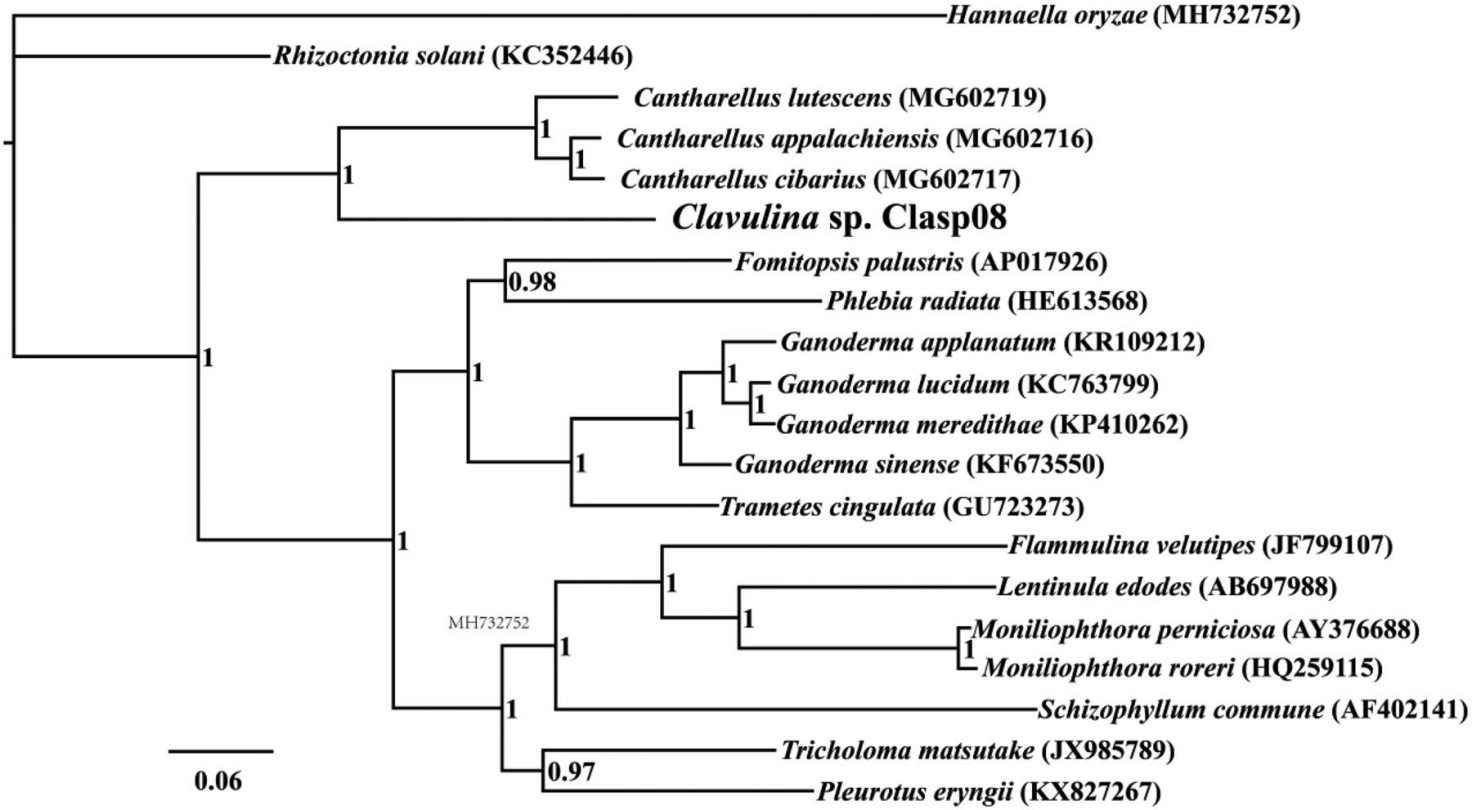
Bayesian phylogenetic analysis of 20 species based on the combined 14 core protein-coding genes. Accession numbers of mitochondrial sequences used in the phylogenetic analysis are listed in brackets after species.

## Data Availability

This mitogenome of *Clavulina* sp. was submitted to GenBank under the accession number of MT649302 (https://www.ncbi.nlm.nih.gov/nuccore/ MT649302).

## References

[CIT0001] Bankevich A, Nurk S, Antipov D, Gurevich AA, Dvorkin M, Kulikov AS, Lesin VM, Nikolenko SI, Pham S, Prjibelski AD, et al. 2012. SPAdes: a new genome assembly algorithm and its applications to single-cell sequencing. J Comput Biol. 19(5):455–477. 22506599 10.1089/cmb.2012.0021 PMC3342519

[CIT0002] Deo GS, Khatra J, Buttar S, Li WM, Tackaberry LE, Massicotte HB, Egger KN, Reimer K, Lee CH. 2019. Antiproliferative, immunostimulatory, and anti-inflammatory activities of extracts derived from mushrooms collected in Haida Gwaii, British Columbia (Canada). Int J Med Mushrooms. 21(7):629–643. 31679298 10.1615/IntJMedMushrooms.2019031193

[CIT0003] Henkel TW, Aime MC, Uehling JK, Smith ME. 2011. New species and distribution records of Clavulina (Cantharellales, Basidiomycota) from the Guiana Shield. Mycologia. 103(4):883–894. 21262982 10.3852/10-355

[CIT0004] Khaund P, Joshi SR. 2014. DNA barcoding of wild edible mushrooms consumed by the ethnic tribes of India. Gene. 550(1):123–130. 25130907 10.1016/j.gene.2014.08.027

[CIT0005] Li Q, Chen C, Xiong C, Jin X, Chen Z, Huang W. 2018. Comparative mitogenomics reveals large-scale gene rearrangements in the mitochondrial genome of two Pleurotus species. Appl Microbiol Biotechnol. 102(14):6143–6153. 29799088 10.1007/s00253-018-9082-6

[CIT0006] Li Q, He X, Ren Y, Xiong C, Jin X, Peng L, Huang W. 2020. Comparative mitogenome analysis reveals mitochondrial genome differentiation in Ectomycorrhizal and asymbiotic *Amanita* species. Front Microbiol. 11:1382. 32636830 10.3389/fmicb.2020.01382 PMC7318869

[CIT0007] Li Q, Liao M, Yang M, Xiong C, Jin X, Chen Z, Huang W. 2018. Characterization of the mitochondrial genomes of three species in the ectomycorrhizal genus *Cantharellus* and phylogeny of Agaricomycetes. Int J Biol Macromol. 118:756–769. 29959010 10.1016/j.ijbiomac.2018.06.129

[CIT0008] Li Q, Ren Y, Shi X, Peng L, Zhao J, Song Y, Zhao G. 2019. Comparative mitochondrial genome analysis of two Ectomycorrhizal fungi (Rhizopogon) reveals dynamic changes of intron and phylogenetic relationships of the subphylum Agaricomycotina. Int J Mol Sci. 20(20):5167. 10.3390/ijms20205167 PMC6829451 31635252

[CIT0009] Li Q, Wang Q, Chen C, Jin X, Chen Z, Xiong C, Li P, Zhao J, Huang W. 2018. Characterization and comparative mitogenomic analysis of six newly sequenced mitochondrial genomes from ectomycorrhizal fungi (Russula) and phylogenetic analysis of the Agaricomycetes. Int J Biol Macromol. 119:792–802. 30076929 10.1016/j.ijbiomac.2018.07.197

[CIT0010] Li Q, Wang Q, Jin X, Chen Z, Xiong C, Li P, Liu Q, Huang W. 2019. Characterization and comparative analysis of six complete mitochondrial genomes from ectomycorrhizal fungi of the *Lactarius* genus and phylogenetic analysis of the Agaricomycetes. Int J Biol Macromol. 121:249–260. 30308282 10.1016/j.ijbiomac.2018.10.029

[CIT0011] Li Q, Wang Q, Jin X, Chen Z, Xiong C, Li P, Zhao J, Huang W. 2019. Characterization and comparison of the mitochondrial genomes from two Lyophyllum fungal species and insights into phylogeny of Agaricomycetes. Int J Biol Macromol. 121:364–372. 30315880 10.1016/j.ijbiomac.2018.10.037

[CIT0012] Li Q, Xiang D, Wan Y, Wu Q, Wu X, Ma C, Song Y, Zhao G, Huang W. 2019. The complete mitochondrial genomes of five important medicinal *Ganoderma* species: features, evolution, and phylogeny. Int J Biol Macromol. 139:397–408. 31381907 10.1016/j.ijbiomac.2019.08.003

[CIT0013] Li Q, Yang L, Xiang D, Wan Y, Wu Q, Huang W, Zhao G. 2020. The complete mitochondrial genomes of two model Ectomycorrhizal fungi (Laccaria): features, intron dynamics and phylogenetic implications. Int J Biol Macromol. 145:974–984. 31669472 10.1016/j.ijbiomac.2019.09.188

[CIT0014] Olariaga I, Jugo BM, Garcia-Etxebarria K, Salcedo I. 2009. Species delimitation in the European species of *Clavulina* (Cantharellales, Basidiomycota) inferred from phylogenetic analyses of ITS region and morphological data. Mycol Res. 113(Pt 11):1261–1270. 19695328 10.1016/j.mycres.2009.08.008

[CIT0015] Thacker JR, Henkel TW. 2004. New species of *Clavulina* from Guyana. Mycologia. 96(3):650–657. 21148885

[CIT0016] Uehling JK, Henkel TW, Aime MC, Vilgalys R, Smith ME. 2012a. New species and distribution records for *Clavulina* (Cantharellales, Basidiomycota) from the Guiana Shield, with a key to the lowland neotropical taxa. Fungal Biol. 116(12):1263–1274. 23245619 10.1016/j.funbio.2012.09.004

[CIT0017] Uehling JK, Henkel TW, Aime MC, Vilgalys R, Smith ME. 2012b. New species of *Clavulina* (Cantharellales, Basidiomycota) with resupinate and effused basidiomata from the Guiana Shield. Mycologia. 104(2):547–556. 22067306 10.3852/11-130

[CIT0018] Wang X, Song A, Wang F, Chen M, Li X, Li Q, Liu N. 2020. The 206 kbp mitochondrial genome of Phanerochaete carnosa reveals dynamics of introns, accumulation of repeat sequences and plasmid-derived genes. Int J Biol Macromol. 162:209–219. 32562727 10.1016/j.ijbiomac.2020.06.142

